# Pharmacognostic standardization of leaves of *Calotropis procera* (Ait.) R. Br. (*Asclepiadaceae*)

**DOI:** 10.4103/0974-7788.59938

**Published:** 2010

**Authors:** Y Murti, B Yogi, D Pathak

**Affiliations:** *Department of Pharmaceutical Chemistry, Rajiv Academy for Pharmacy, Mathura, N.H. #2, Delhi-Mathura Bye-pass, P.O. Chattikara, Mathura - 281 001, (U.P.), India*

**Keywords:** *Calotropis procera* leaves, fluorescence analysis, macroscopy, microscopy, pharmacognostic standardization, phytochemical screening

## Abstract

*Calotropis procera*, belonging to the Asclepidaceae family, is present more or less throughout India and in other warm, dry places such as, Warizistan, Afghanistan, Egypt, and tropical Africa. Its common names are Akra, Akanal, and Madar. The leaves of *Calotropis procera* are said to be valuable as an antidote for snake bite, sinus fistula, rheumatism, mumps, burn injuries, and body pain. The leaves of *Calotropis procera* are also used to treat jaundice. A study on *Calotropis procera* leaf samples extracted the air-dried leaf powder with different solvents such as petroleum-ether (60-80°C), benzene, chloroform, ethanol, and sterile water. Preliminary phytochemical analysis was done long with measurement of the leaf constants, fluorescence characteristics, and extractive values. Quantitative estimation of total ash value, acid insoluble ash, and water- soluble ash may serve as useful indices for identification of the powdered drug. Histochemical studies which reveal rows of cylindrical palisade cells and, vascular bundles may also serve as useful indices for identification of the tissues. These studies suggested that the observed pharmacognostic and physiochemical parameters are of great value in quality control and formulation development of *Calotropis procera*.

## INTRODUCTION

The plant, *Calotropis procera* (of family *Asclepiadaceae*), commonly known as Ak is used in many ayurvedic formulations like *Arkelavana*. The medicinal potential of *Calotropis procera* has been known to traditional systems of medicine for a while now with its leaves being widely used. The use of the plants, plant extracts, and pure compounds isolated from natural sources has always provided a foundation for modern pharmaceutical compounds. [1] *Calotropis procera* is a well known plant and has been traditionally used for diarrhoea, stomatic, sinus fistula, and skin disease,[2,3] and the leaf part is used to treat jaundice. There are many reports on the geographical distribution, habitat, and morphological characters of the plant. However, no work has been carried out on the leaves of this plant, which contain potentially useful ethnomedicinal drugs. Therefore, the present work was undertaken to study the pharmacognostic aspects of *Calotropis procera* leaves.

## MATERIALS AND METHODS

### Plant materials

Fresh leaves were collected from the *Calotropis procera* plant growing in the medicinal garden of Rajiv Academy for Pharmacy, Mathura, U.P., India. The plant specimens were authenticated (voucher specimen: NISCAIR/RHMD/ Consult/-2008-09/1144/176) and the leaves were washed in running water and air-dried. The fresh leaf was then studied for pharmacognostic evaluation, including examination of morphological and microscopic characteristics and some preliminary phytochemical evaluation.

### Instrumentation and techniques

Leaf specimens were cut into rectangular pieces that included the midrib and a portion of the lamina. For paradermal sections, specimens measuring 0.05 cm^2^ were cut out from the midrib portion of the lamina. The leaf specimens were fixed and embedded in paraffin blocks,[4] followed by dehydration, infiltration, and sectioning,[5] and finally staining and photographing of the sections.[6] Photography was done by using a Nikon Labphot 2 microscopic unit. Descriptive features were matched with those included in standard anatomical books.[7,8] Air-dried leaves were powdered using a homogenizer and the leaf powder was considered as drug. The leaf powder and the extracts of the powder in different solvents were examined under ordinary day light and in UV-light (254 nm). The fluorescence was determined according to the methods of Chase and Pratt. [9] The total ash, water-soluble ash, and acid-insoluble ash content was determined by employing standard methods of analysis as described[10] in the Indian Pharmacopoeia (1966). Quantitative determinations of the powdered drug like physicochemical constants,[11] fluorescence,[12] and a preliminary phytochemical screening[13,14] were carried out.

### Morphological characteristics of leaf

**Figure 1:**
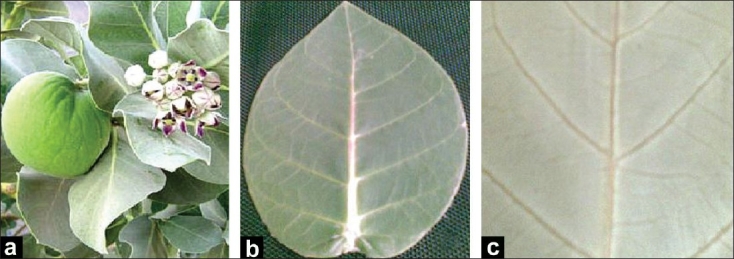
Morphology of *Calotropis procera* leaf [a - A twig with oppositely arranged subsessile leaves; b - Broadly ovate or elliptical, cottony, pubescent when young and glabrous on maturity; c - Portion of the lamina showing venation pattern]

*Calotropis* is a large, bushy shrub with decussate, obovate, coriaceous, auriculate, leaves with acute, subsessile apices extraaxillary, umbellate, panicale inflorescene with purple corolla and erect lobes.[15–17] The morphological studies revealed the leaves to be subsessile, 6-15 cm by 4.5-8 cm, broadly ovate, ovate-oblong, elliptical, or obovate, acute, pubescent when young and glabrous on both sides on maturity [Figure 1].

### Microscopical characteristics of the leaf

Transverse sections through the midrib showed an upper and lower, single- layered epidermis that was externally covered with a thick, striated cuticle, a few epidermal cells on both lower and upper surfaces, parenchymatous cells that were thin-walled and isodiametric to circular. Intracellular spaces were present in ground tissue and the stele was crescent-shaped and composed of bicollateral and open vascular bundles. The xylem consisted mostly of vessels and tracheids, and a strip of cambium was present between the xylem and phloem tissues. Laticifers were also present along with the phloem and parenchymatous zone.

**Figure 2:**
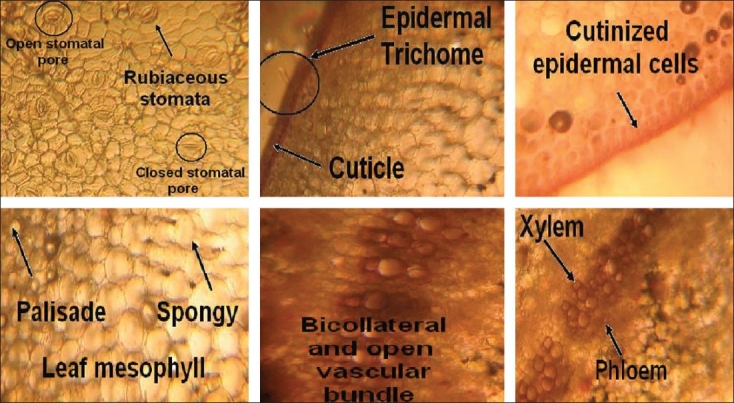
Microscopy of *Calotropis procera* leaf

The lamina which was dorsiventral with the mesophyll, was seen to be differentiated into a palisade and spongy tissue. The upper and lower epidermise were covered externally with a thick, striated cuticle. Below the upper epidermis were three rows of elongated, closely arranged, palisade parenchyma. Spongy parenchyma tissues were almost radially elongated with intracellular spaces. Central cells were irregular in shape; laticifers and vascular bundles were also present scattered in this region; the details are shown in Figure 2.

## RESULTS

The present study highlights the results of a comprehensive study on the microscopic parameters including the gross anatomical features, leaf constants, cellular composition, tissue organization, and cellular inclusion of the leaf. The lamina which was dorsiventral with the mesophyll, was seen to be differentiated into a palisade and spongy tissue. The upper and lower epidermis were covered externally with a thick, striated cuticle. Below the upper epidermis were three rows of elongated, closely arranged, palisade parenchyma. Spongy parenchyma tissues were almost radially elongated with intracellular spaces. Central cells were irregular in shape; laticifers and vascular bundles were also present scattered in this region; the details are shown in Figure 2.

The results of the quantitative and qualitative analysis of the leaf samples are depicted in Tables 1–4.

**Table 1:** Leaf constants of *Calotropis procera* leaf[18]

Leaf constant	Value
Stomatal index	44
Stomatal number	11
Palisade ratio	14.8
Vein-islet number	25.2
Veinlet termination number	19.7

**Table 2:** Ash value of powdered leaf of *Calotropis procera*

Type of ash	Ash value (%)
Total ash	18.3
Acid-insoluble ash	1.6
Water-soluble ash	1.9

**Table 3:** Fluorescence of *Calotropis procera* leaf powder in different solvents

Treatment	Under visible light	U.V. light (short wavelength; 254 nm)
Powder as such	Green	No change
Powder + 1N NaOH	Light green	Green
(aqueous)		
Powder + 1N NaOH	Pale green	Light green
(ethanolic)		
Powder + 1N HCl	Green	Green
Powder + 50% HNO_3_	Brown	Green

**Table 4:** Preliminary phytochemical screening of leaf powder of *Calotropis procera*

Phyto chemicals	Petroleum ether extracts (60-80°C)	Chloroform extracts	Ethanol extracts	Water extract
Alkaloids	−	−	−	−
Sugars	−	−	+	−
Phenols	−	+	+	+
Flavonoids	−	−	+	−
Saponins	−	−	−	+
Steroids	+	−	+	−
Terpenoids	−	+	+	−
Tannins	+	−	+	+
Fatty acids	−	−	−	−
Glycosides	+	+	+	+

‘+’ = Presence of the compound; ‘−’ = Absence of compound

## DISCUSSION

By virtue of their photosynthetic machinery, leaves serve as a sink for several metabolites and as an important source of several bioactive compounds. The macroscopic and microscopic evaluation of leaves of *Calotropis procera*, the quantitative estimation of leaf constants, ash values, and fluorescence, and preliminary phytochemical screening of the leaf powder would be of considerable use in the identification of this drug. Empirical knowledge about medicinal plants plays a vital role in primary health care and has great potential for the discovery of new herbal drugs. These findings may be useful to supplement existing information with regard to the identification and standardization of *Calotropis procera*, even in the powdered form of the plant drug, to distinguish it from substitutes and adulterants. These studies also suggested that the observed pharmacognostic and physiochemical parameters are of great value in quality control and formulation development. In conclusion, the present study may be useful to supplement information with regard to its identification and, standardization, and in carrying out further research and revalidation of its use in the Ayurvedic System of Medicine.
